# Carbohydrate metabolism during vertebrate appendage regeneration: What is its role? How is it regulated?: A postulation that regenerating vertebrate appendages facilitate glycolytic and pentose phosphate pathways to fuel macromolecule biosynthesis

**DOI:** 10.1002/bies.201300110

**Published:** 2013-11-22

**Authors:** Nick R Love, Mathias Ziegler, Yaoyao Chen, Enrique Amaya

**Affiliations:** 1) Department of Molecular Biology, University of BergenBergen, Norway; 2) The Healing Foundation Centre, Faculty of Life Sciences, University of ManchesterManchester, UK; 3) Laboratory for Organogenesis and Neurogenesis, RIKEN Center for Developmental BiologyChuo-Ku, Kobe, Japan; 4) Wellcome Trust – Medical Research Council Cambridge Stem Cell Institute, University of CambridgeCambridge, UK

**Keywords:** genetically encoded indicator, glycolysis, metabolism, pentose phosphate pathway, tissue regeneration, Warburg effect, *Xenopus* tadpole tail regeneration

## Abstract

We recently examined gene expression during *Xenopus* tadpole tail appendage regeneration and found that carbohydrate regulatory genes were dramatically altered during the regeneration process. In this essay, we speculate that these changes in gene expression play an essential role during regeneration by stimulating the anabolic pathways required for the reconstruction of a new appendage. We hypothesize that during regeneration, cells use *leptin*, *slc2a3*, *proinsulin*, *g6pd*, *hif1*α expression, receptor tyrosine kinase (RTK) signaling, and the production of reactive oxygen species (ROS) to promote glucose entry into glycolysis and the pentose phosphate pathway (PPP), thus stimulating macromolecular biosynthesis. We suggest that this metabolic shift is integral to the appendage regeneration program and that the *Xenopus* model is a powerful experimental system to further explore this phenomenon.

## Introduction

Vertebrate appendage regeneration entails the reconstruction of outward growing tissue structures, including limbs, fins, digits, and tails. Many vertebrate species, including fish, amphibians, and reptiles, and to a lesser extent mammals, have the ability to regenerate their appendages following amputation 1,2 (for an example of vertebrate tail appendage regeneration, see Supplementary Movie 1). The regeneration process coordinates a variety of biological processes, all of which rely on molecules and energetic equivalents produced during cellular metabolism. Yet despite its intuitive importance, very little is known about how cellular metabolism is regulated during vertebrate tissue regeneration.

Tissue regrowth during appendage regeneration is an inherently anabolic process. Cells of regenerating tissues must alter their metabolic program in order to accommodate the increased production of new cell membranes, proteins, and nucleic acids. Most biosynthetic pathways require carbon-containing precursor molecules generated directly or indirectly (though not exclusively) from carbohydrates such as glucose. For this reason, glucose utilization can be viewed as a convenient starting point to better understand the greater metabolic network utilized during appendage regeneration.

We recently found that the expression of a substantial number of genes governing glucose metabolism was greatly altered during *Xenopus* tadpole tail regeneration 3. These data and others have led us to hypothesize that glucose metabolism and its regulation plays an essential role during vertebrate appendage regeneration. Here we take the opportunity to highlight the largely ignored role for carbohydrate metabolism during appendage regeneration and to encourage research aimed at better linking these two processes.

## The phases of *Xenopus* tail appendage regeneration

**Figure 1:**
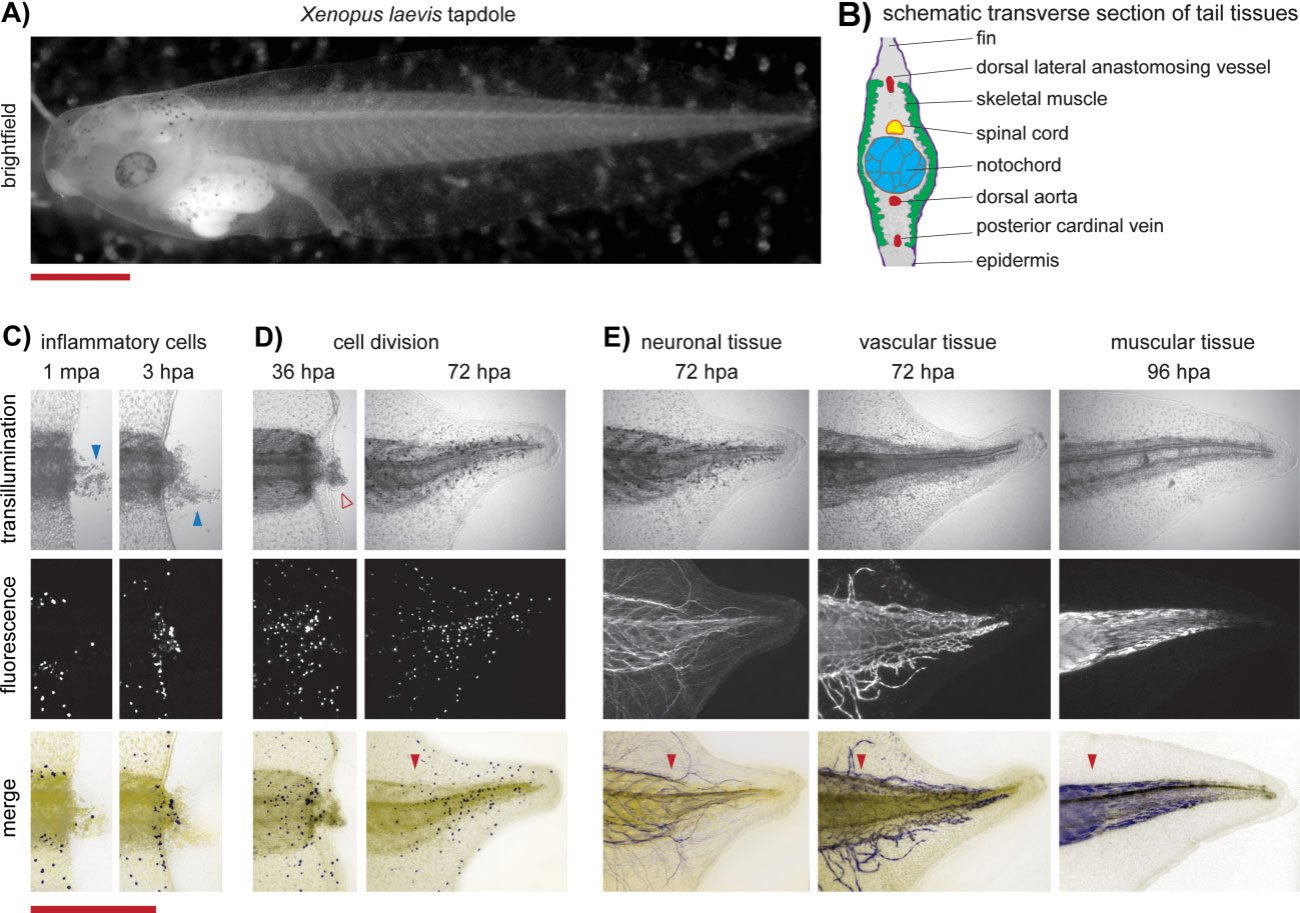
Tissue regrowth during *Xenopus* tadpole tail appendage regeneration. A: *Xenopus laevis* tadpole. Scale bar represents 500 µm. B: Schematic diagram of a transverse section of the tadpole tail. C: Transillumination and fluorescence images showing the recruitment of inflammatory cells to the amputation site by 3 hours post-amputation (hpa). Blue arrow shows blood and other cellular debris that spilled from the wound site by 1 minute post-amputation (mpa). Fluorescence signal detects inflammatory cells using a *Xenopus laevis* transgenic line 42. Scale bar represents 500 µm and is applicable to the panels in D and E. D: Transillumination and immunofluorescence (anti-phosphohistone H3) images showing proliferating cells at two different time periods during *Xenopus laevis* tail regeneration. Open red arrow shows regenerative bud tissue. E: Transillumination and immunofluorescence images showing the regeneration of neuronal tissue (anti-*N*-acetylated tubulin), vascular tissue (Flk-1: eGFP *X. laevis* transgenic line 45, and skeletal muscle (anti-12/101, 46).

The *Xenopus* tadpole tail contains a diverse collection of axial tissues, including the spinal cord, dorsal aorta, notochord, skeletal muscle, and epidermis (Fig. 1A and B) (3, reviewed in 4). All of these tissues regenerate within one week following tail amputation. Elegant grafting experiments have shown that most of the regenerated tail tissues are derived from lineage specific precursors 5. In the case of skeletal muscle, tail amputation activates stem cell-like muscle satellite cells, which then differentiate and repopulate the skeletal muscle of the new tail 5. Several growth factors govern tail regeneration, including the BMP, Notch, Wnt, Fgf, and TGFβ pathways 6–8.

*Xenopus* tadpole tail regeneration can be divided into three phases: an early, intermediate, and late phase 3. During the early phase (from 0 to 24 hours post-amputation (hpa)), epidermal wound healing occurs, and inflammatory cells migrate to the site of injury (Fig. 1C). During the intermediate phase, (from ∼24 to 48 hpa), a regenerative tissue bud appears distal to the injury site and an increased rate of cell proliferation becomes apparent (Fig. 1D). During the late phase (from ∼48 hpa onwards), the tail and its tissues (including blood vessels, neurons, and muscle) regenerate to reconstitute a fully functional appendage (Fig. 1E).

## The expression of glucose import modulators increases during *Xenopus* tadpole tail appendage regeneration

To better understand *Xenopus tropicalis* tadpole tail regeneration, we decided to identify which genes changed their expression levels during the regenerative response. To do this, we collected RNA samples from the early, intermediate, and late phases of regeneration (as well as a pre-amputation reference) and analyzed them using genome-wide Affymetrix microarrays (MIAME Experiment E-MEXP-2420) 3. We found that the most highly upregulated gene following tail amputation was *leptin*, a gene that encodes a cytokine that regulates appetite and blood vessel growth 3,9,10. The gene expression data also showed that *proinsulin*, the gene that encodes insulin, was upregulated approximately threefold following tail amputation.

Both leptin and insulin stimulate glucose import into cells by increasing the activity of glucose transporters 11. These transporters are composed of many different subunits 12, and genes encoding some of these subunits were also markedly upregulated following tail amputation. An example is the expression level of *slc2a3* (*facilitated glucose transporter*, *member 3)*, which was elevated 25-fold within six hours following amputation.

In addition, some of the signaling pathways implicated during tail regeneration can alter cellular glucose metabolism and intake. For example, PI3K/Akt signaling has been shown to increase glucose transport into cells and activate the glucose metabolic enzymes hexokinase (HK) and phosphofructokinase (PFK) 13,14. Notably, both leptin and insulin activate PI3K/Akt signaling, as do several receptor tyrosine kinase (RTKs) that have been implicated in tail regeneration 3,9,15.

Together, these data led us to speculate that regenerating tissues actively increase glucose cellular import. Because regeneration is an inherently anabolic process, we reasoned that increased glucose import is important for the production of new macromolecular components. In the next sections, we speculate in more detail on the mechanisms by which glucose metabolism might be utilized and regulated during regeneration.

## Cutting carbon emissions via glycolysis

**Figure 2:**
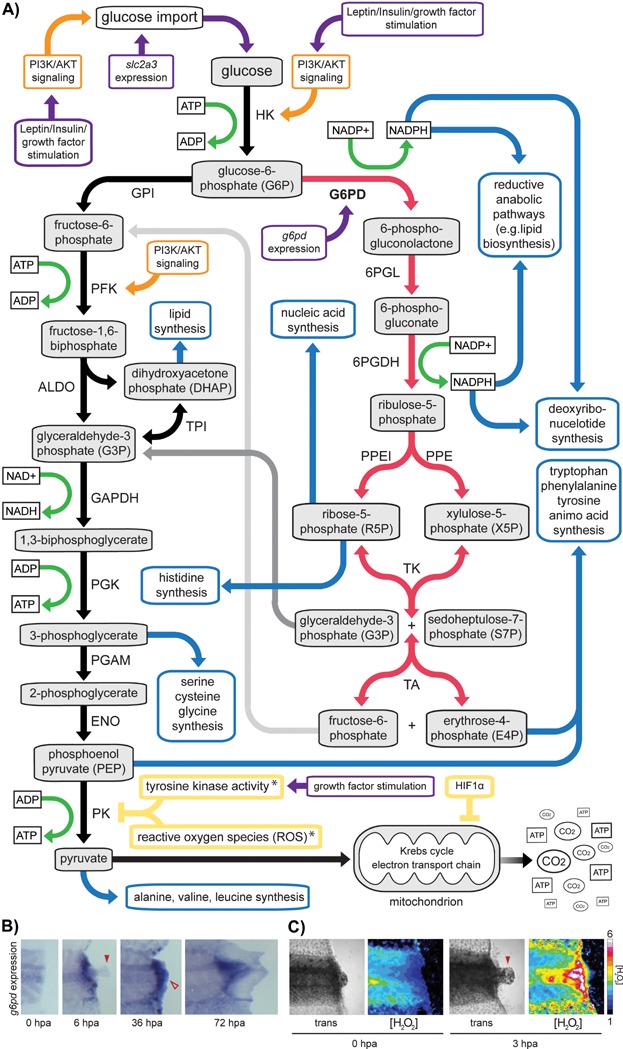
Production of biosynthetic precursors during glycolytic metabolism and their putative regulation during *Xenopus* tail appendage regeneration. A: Pathways demonstrating how glucose or its derivatives can contribute to biosynthetic processes as well as how glucose metabolism may be regulated during appendage regeneration as outlined in the essay. Diagram adapted from 13,16,24. Colors indicate conceptually different pathways or interactions: glycolysis toward glucose combustion (black); pentose phosphate pathway (PPP, shown in red); molecular contributions of biosynthetic pathways (blue); NAD/H, NADP/H, ATD/P reactions shown in green; reintroduction of PPP products into glycolysis (gray); putative inhibitory mechanisms during *Xenopus* tadpole tail regeneration (yellow); putative activation mechanisms during *Xenopus* tadpole tail regeneration (purple); putative activity of PI3/Akt given its previously characterized interactions with leptin/insulin/RTK activity 9,15. Asterisk (*) indicates that the PK inhibition by ROS and tyrosine kinase activity have been reported for the PKM2 version of the PK enzyme 19,20. Acronyms are as follows: HK, hexokinase; G6PD, glucose-6-phosphate dehydrogenase; 6PGL, 6-phosphogluconolactonase; 6PGDH, 6-phosphogluconate dehydrogenase; PPEI, phosphopentoseisomerase; PPE, phosphopentose epimerase; PFK, phosphofructokinase; TK, transketolase; TA, transaldolase; PGI, phosphoglucose isomerase; ALDO, aldolase; TPI, triosephosphate isomerase; GAPDH, glyceraldehyde phosphate dehydrogenase; PGK, phosphoglycerate kinase; PGAM, phosphoglycerate mutase; ENO, enolase; PK, pyruvate kinase. B: In situ hybridization showing expression of *g6pd* following amputation and during the regeneration of *Xenopus tropicalis* tadpole tails. Solid red arrow shows a portion of the notochord that has exited the wound site. Open red arrow shows regenerative bud tissue. C: Transillumination (trans) and HyperYFP ([H_2_O_2_]) images showing the detection of the reactive oxygen species (ROS) hydrogen peroxide (H_2_O_2_) following *Xenopus laevis* tail amputation using the H_2_O_2_ sensitive HyPerYFP probe 21,42. Relative levels of H_2_O_2_ levels shown in the scale found to the right of the images. Solid red arrow shows a portion of the notochord that has exited the wound site.

During its complete combustion, glucose generates approximately 36 energy-bearing ATPs and six CO_2_ molecules (Fig. 2). However, from the viewpoint of a rapidly growing tissue system, CO_2_ “emissions” can be considered detrimental, as molecular carbon substrates are needed for the anabolic reactions that underlie tissue growth 14. Toward this aim, inhibiting the complete combustion of glucose (and thus the generation of CO_2_) allows glucose to be diverted into anabolic pathways that generate nucleic acids, proteins, and lipids (Fig. 2A).

During its complete combustion, glucose is first processed in glycolysis, generating two molecules of pyruvate that are later fully oxidized in the Krebs cycle (Fig. 2A). However, instead of entering the Krebs cycle, glucose derivatives produced in glycolysis can be used in anabolic biosynthetic reactions (Fig. 2A) 16. For instance, dihydroxyacetone phosphate (DHAP) can be used in the production of certain lipids; and 3-phosphoglycerate and pyruvate can be used in the synthesis of several amino acids, such as serine, cysteine, glycine, alanine, valine, and leucine, thus contributing to an increase in protein mass.

Other macromolecular precursors and co-factors are generated in the pentose phosphate pathway (PPP), a metabolic pathway that stems from glucose after its phosphorylation by HK (Fig. 2A). The rate-limiting step of glucose-6-phosphate entry into the PPP is governed by the enzyme, glucose-6-phosphate dehydrogenase (G6PD) 17. We found that *g6pd*, the gene that encodes G6PD, was significantly upregulated within six hours following amputation and remained at high levels throughout the intermediate and late phases of regeneration 3 (Fig. 2B), suggesting that the PPP is promoted during tissue regeneration.

Oxidation reactions in the PPP generate two molecules of NADPH, a co-factor, which is critical for lipid synthesis (Fig. 2A). NADPH is also essential for the production of the deoxyribonucleotides needed for DNA synthesis (Fig. 2). Moreover, the PPP generates ribose-5-phosphate (R5P), which is essential for the production of nucleic acids and the amino acid histidine. Finally, the PPP gives rise to erythrose-4-phosphate (E4P), which when combined with phosphoenol pyruvate (PEP), is involved in the generation of the aromatic amino acids tyrosine, phenylalanine, and tryptophan. These observations suggest that an increase in glucose entry into glycolysis, combined with shunting of glucose into the PPP through the upregulation of *g6pd* expression, may play crucial roles in facilitating the regeneration program.

## ROS sensitive pyruvate kinase isoform 2 (PKM2) controls carbohydrate flux from glycolysis into the Krebs cycle

In order for glucose to be used in glycolysis and the PPP, its entry into the Krebs cycle should be diminished. A well-studied enzyme that controls the flow of glucose into the Krebs cycle is pyruvate kinase M (PKM). This enzyme mediates the conversion of PEP to pyruvate in the final step of glycolysis (Fig. 2A). PKM therefore regulates the balance between glycolysis and oxidative phosphorylation. Two differentially spliced isoforms of the *pkm* gene have been described, dubbed *pkm1* and *pkm2*. Of particular interest is PKM2, which is highly expressed in embryonic and cancer tissues 18.

PKM2 activity can be inhibited by growth factor stimulated tyrosine phosphorylation 19. This is relevant because pathways that activate RTKs, such as the FGF signaling, are known to be necessary during *Xenopus* tail appendage regeneration 6. Also, reactive oxygen species (ROS) have been shown to inhibit the activity of PKM2 via the oxidation of one of its cysteine residues 20. Notably, we have found that ROS production is markedly increased and required for *Xenopus* tadpole tail regeneration (Fig. 2C) 21.

Our gene expression data also showed that injured *Xenopus* tail tissues increase the level of expression of *hif1*α 3, which has been shown to suppress the metabolic activities of mitochondria 22. Thus, we hypothesize that tyrosine phosphorylation, ROS production, and *hif1*α expression coordinately play essential roles in decreasing the combustion of glucose during appendage regeneration and thus increase carbohydrate entry into the anabolic pathways necessary for tissue growth.

## Glucose utilization in proliferating systems: The Warburg effect

Previous studies have shown that rapidly dividing tissues, such as tumors, exhibit altered metabolism and glucose utilization 23,24. In the 1920s, Nobel laureate Otto Warburg reported that, even in the presence of sufficient oxygen, cancerous tissue exhibits decreased oxygen consumption per catabolized glucose molecule, a phenomenon known as the Warburg effect or aerobic glycolysis 24,25. In other words, cancer cells increase glucose consumption to maximize biosynthetic capacity rather than enhance their ATP supply via pyruvate oxidation in the Krebs cycle.

Warburg's initial observation was later confirmed in experiments examining proliferating lymphocytes, suggesting that increased glycolysis could be somewhat inherent to rapidly dividing cells 26. Recently, a Warburg effect has also been described in proliferating embryonic tissues 27–29. Stem cells may also depend on Warburg-like metabolism. Recent evidence suggests that induction of pluripotency in differentiated cells correlates with a shift to a more glycolytic state 30,31. Whether the muscle satellite stem cells implicated during *Xenopus* tadpole tail regeneration depend on Warburg-like metabolism is an intriguing possibility to be examined in future studies.

Studies have also shown that PPP dependent processes – such as NADPH-dependent detoxifying mechanisms and production of reactive oxygen species (ROS) – are implicated during cancer progression 32,33. Accordingly, genetic studies have reported that cancerous tissues exhibit increased expression of glycolytic enzymes 34.

The abnormally high rate of glucose uptake and glycolysis in cancerous tissues has prompted glycolytic pathway inhibitors to be explored as anti-cancer agents 35. In addition, the radioactively labeled glucose substrate analog fluorodeoxyglucose (FDG) is currently used to help locate cancers within the body using positron emission tomography (PET) 36.

These studies demonstrate that altered glucose metabolism – the Warburg effect – can be viewed as a general property of proliferating systems. Although it has never been formally reported, we argue here that a Warburg-like metabolism may be an essential property of regenerating tissues.

## Using the *Xenopus* model to examine carbohydrate metabolism during vertebrate appendage regeneration

Thus far we have discussed how gene expression (*leptin*, *proinsulin*, *slc2a3*, *g6pd*, *hif1*α), signaling pathways (PI3K/Akt signaling downstream of leptin/insulin/PDGF, PKM2 inhibition downstream of RTK activity), and the production of ROS (ROS sensitive PKM2 inhibition) are implicated during tail regeneration. We have hypothesized that these collectively function to increase carbohydrate flux into anabolic reactions. Given that tissue regrowth is biosynthetic in nature, the idea that glucose metabolism is altered during regeneration to accommodate anabolic pathways makes sense. However, these ideas have not been formally tested.

We would argue that the *Xenopus* tadpole tail regeneration model represents an ideal system to investigate the role and regulation of carbohydrate metabolism during appendage regeneration. The *Xenopus* model has a well-developed series of genomic resources, such as a sequenced genome 37 and over one million ESTs 38. Frogs are relatively easy to house, and tadpoles can be raised in the thousands at minimal cost 39. The tadpole tail is semi-transparent, allowing live imaging of regenerating tissues. Furthermore, the *Xenopus* model is amenable to a wide range of genetic modification protocols, including targeted mutations 40 and the generation of transgenic lines 41.

Genetic modification of *Xenopus* can be exploited to address how glucose is utilized and regulated during regeneration. For example, to determine whether leptin signaling is important for proper appendage regeneration, one can generate gene knockouts of the *leptin* gene in *Xenopus*, using targeted genome editing technology such as activator-like effector nucleases (TALENs) 40.

In addition, transgenic *Xenopus* lines can be produced to allow the analysis of metabolic changes during regeneration in vivo, over long periods of time, and in a tissue-specific manner. For example, in a previous study we generated transgenic *Xenopus* lines that ubiquitously expressed a ROS-sensitive molecular sensor, called HyperYFP 42. This transgenic line allowed us to assess the changes in ROS levels during tail regeneration (Fig. 2C) 21. A similar approach can be exploited in order to generate additional transgenic lines that express genetically encoded fluorescent metabolic indicators. One such tantalizing genetically encoded indicator is the Peredox protein, a GFP-RFP fusion protein that reports changes in NAD^+^/NADH ratios, a major readout of cellular metabolism 43.

Aside from genetic modification, experiments using *Xenopus* tadpoles could also address questions regarding glucose intake during appendage regeneration. One particularly intriguing experiment, if feasible, would be to subject a regenerating organism to food or culture medium supplemented with FDG and subsequently performing FDG-PET on the regenerating organism – much like the PET scans of cancer patients – in order to assess whether an increase in glucose uptake occurs during tissue regeneration.

Similarly, experiments using *Xenopus* tadpoles could address whether regenerating appendage tissues exhibit the Warburg effect. A straightforward way to assess this possibility would be to replicate similar experiments to those performed by Otto Warburg and others on regenerating tissues. In addition, assessing the activity of glycolytic enzymes such as PK during regeneration would also provide evidence of increased carbon flux into glycolysis. This approach has previously been done in regenerating rat liver 44. Performing metabolomic analyses on regenerating appendages, such as tails and limbs, could further corroborate such studies.

Additional experiments could determine whether the glycolysis-promoting isoforms of PKM, such as PKM2, are preferentially expressed in regenerating tissues. In addition, examining the phosphorylation or oxidation state of the PKM2 via Western blot or targeted proteomic analyses might also help elucidate whether PK activity is modulated during different phases of regeneration. These experiments might help confirm whether anabolic pathways are promoted at the expense of oxidative phosphorylation.

## Conclusions and prospects

Vertebrate appendage regeneration is a fascinating process that is not yet fully understood. In particular, we know little about how cells alter their cellular metabolism during regeneration. Here we have used recent evidence to speculate that regenerating appendages utilize several mechanisms to shift glucose metabolism toward anabolic pathways. Confirming these speculations may be an important step toward the development of more effective regenerative therapies, as proper cellular metabolism may facilitate a more efficient regenerative response.

In this regard, the *Xenopus* tadpole model is a powerful system to investigate the metabolic components of vertebrate appendage regeneration. However, discoveries made in *Xenopus* should be confirmed in other models of appendage regeneration – including zebrafish fin regeneration, mouse digit regeneration, and limb regeneration in the Mexican salamander/axolotl (*Ambystoma mexicanum*) – before a more complete understanding of the role and regulation of carbohydrate metabolism during vertebrate appendage regeneration can emerge. Indeed, investigations using these models may potentially yield insights into the fundamental and evolutionarily conserved metabolic underpinnings of successful vertebrate appendage regeneration, and may even shed light into why some organisms have better regenerative capacities than others.

## Abbreviations

ALDO: aldolase
ENO: enolase
FDG: fluorodeoxyglucose
GAPDH: glyceraldehyde phosphate dehydrogenase
G6PD: glucose-6-phosphate dehydrogenase
HK: hexokinase
hpa: hours post-amputation
PET: positron emission tomography
PFK: phosphofructokinase
PGI: phosphoglucose isomerase
PGK: phosphoglycerate kinase
PGM: phosphoglycerate mutase
PKM1: pyruvate kinase isoform 1
PKM2: pyruvate kinase isoform 2
PPP: pentose phosphate pathway
ROS: reactive oxygen species
TA: transaldolase
TALENs: transcription activator-like effector nucleases
TK: transketolase
TPI: triosephosphate isomerase
